# Construction of cytogenetic map of *Gossypium herbaceum* chromosome 1 and its integration with genetic maps

**DOI:** 10.1186/s13039-015-0106-y

**Published:** 2015-01-22

**Authors:** Xinglei Cui, Fang Liu, Yuling Liu, Zhongli Zhou, Yanyan Zhao, Chunying Wang, Xingxing Wang, Xiaoyan Cai, Yuhong Wang, Fei Meng, Renhai Peng, Kunbo Wang

**Affiliations:** State Key Laboratory of Cotton Biology (China)/Institute of Cotton Research of Chinese Academy of Agricultural Science, Anyang, Henan 455000 China; Anyang Institute of Technology, Anyang, Henan 455000 China

**Keywords:** Cotton, BAC-FISH, Cytogenetic map

## Abstract

**Background:**

Cytogenetic map can provide not only information of the genome structure, but also can build a solid foundation for genetic research. With the developments of molecular and cytogenetic studies in cotton (*Gossypium*), the construction of cytogenetic map is becoming more and more imperative.

**Results:**

A cytogenetic map of chromosome 1 (A_1_01) of *Gossypium herbaceum* (A_1_) which includes 10 bacterial artificial chromosome (BAC) clones was constructed by using fluorescent *in situ* hybridization (FISH). Meanwhile, comparison and analysis were made for the cytogenetic map of chromosome 1 (A_1_01) of *G. herbaceum* with four genetic linkage maps of chromosome 1 (A_h_01) of *G. hirsutum* ((AD)_1_) and one genetic linkage map of chromosome 1 of (A_1_01) *G. arboreum* (A_2_). The 10 BAC clones were also used to be localized on *G. raimondii* (D_5_) chromosome 1 (D_5_01), and 2 of them showed clear unique hybridized signals. Furthermore, these 2 BAC clones were also shown localized on chromosome 1 of both A sub-genome and D sub-genome of *G. hirsutum*.

**Conclusion:**

The comparison of the cytogenetic map with genetic linkage maps showed that most of the identified marker-tagged BAC clones appearing same orders in different maps except three markers showing different positions, which might indicate chromosomal segmental rearrangements. The positions of the 2 BAC clones which were localized on A_h_01 and D_h_01 chromosomes were almost the same as that on A_1_01 and D_5_01 chromosomes. The corresponding anchored SSR markers of these 2 BAC clones were firstly found to be localized on chromosome D_5_01 (D_h_01) as they were not seen mapped like this in any genetic map reported.

## Background

Allopolyploid formation in plants simply reflects promiscuity of plants or provides a selective advantage in survival has been long debated [1]. Most angiosperm (approximately 70 percent) was thought to have incurred one or more polyploidization events in their history [2]. The genus *Gossypium*, which comprises of 52 species had been classified into 8 diploid (2n = 2× = 26) genomes, i.e. A, B, C, D, E, F, G, and K, and as well one allotetraploid (2n = 4× = 52) genome, i.e. AD [3,4]. In the early stages of the genus evolution, A genome diploids and D genome diploids diverged, acquiring a 2-fold difference in genome size subsequently. The genome-size difference is thought probably associated with the expansion and contraction of repetitive elements including transposons, whereas the homoeologous sequences flanking the genes are highly conserved [5-7]. These two divergent genomes later became reunited with allopolyploid formation approximately 1–2 million years ago (MYA) [8], in the New World by showing hybridization between a maternal Old World “A” genome taxon resembling *Gossypium herbaceum* (2n = 2× = 26) and a paternal New World “D” genome taxon resembling *Gossypium raimondii* or *Gossypium gossypioides* (both 2n = 2× = 26) [9,10]. It is old enough for sequence divergence but relatively recent to maintain genome stability [11]. This proves that cotton (*Gossypium spp*) is not only an important economic plant, but also an excellent system for study on genomic organization, genome-size variation, genome evolution and polyploidization in plants. Therefore, due to its importance in scientific research, sequencing the cotton genome and carrying out genomic research are highly necessary.

The genetic linkage map, which can indicate the orders of markers on chromosomes, is a powerful molecular tool to dissect the genome. Nevertheless, the exact cytogenetic positions of the genetic loci and genomic sequences on the chromosomes shall be not accurately identified by genetic linkage map, due to the unequally distributed crossovers on chromosome arms. Thus loci physically far apart on chromosomes can be linked tightly on genetic linkage maps and *vice versa* [12]. Cytogenetic map, which accurately represent direct inspection of distinctive loci on chromosomes, can compensate for the disadvantage of genetic linkage map. It not only can provide information on the structure and evolution of genomes but also is useful in the synteny comparison between relative genomes, especially for complex-genome organisms which has large amounts of repetitive DNA, such as maize and wheat [13]. So integrating genetic linkage map with cytogenetic map can provide new insight in chromosome structure. However, cytogenetic maps are nascent and relatively underdeveloped in many plants especially in cotton, despite the long history of cytology [14]. Thus, the majority of the genetic linkage maps of cotton were not integrated with any type of physical map.

Fluorescence *in situ* hybridization (FISH), which has been developed from the probe of highly repeated copies sequences to single-copy probe [15,16], and from single-color to multiple-color [17] in recent years, and involving hybridization of labeled DNA fragments to intact chromosomes to show positions of complementary sequences [18-20], shows a good method to construct cytogenetic map. The cytogenetic positions of the associated molecular markers can be then determined accurately and effectively. To date, high-resolution cytogenetic maps of individual chromosomes had been constructed in many crops, such as maize [21,22], rice [13], Brassica [23,24], tomato [25-27], cucumber [12,28], soybean [29], papaya [30], potato [17,31,32], common bean [33,34], and sorghum for all chromosomes [35].

Though the application of FISH in cotton has lagged behind other crops, the development of target DNA has greatly improved its resolution and promoted its application in cytogenetic study [36,37]. Chromosomes of many cotton species were identified and many researches of genome structure of cotton were achieved by using FISH [38-40]. Cytogenetic maps of cotton, in which A_h_12 and D_h_12 homologous chromosomes including 15 and 21 SSR-derived BACs, respectively, had also been developed, and the integration between cytogenetic maps and genetic linkage maps has been comprehensively analyzed [11]. However the cytogenetic maps of cotton are far from completed. In this paper, a cytogenetic map of chromosome 1(A_1_01) of *G. herbaceum* including 10 BAC clones was constructed by using BAC-FISH mapping method and as well the relationship between the cytogenetic map and genetic linkage maps has been analyzed. Furthermore 2 of these 10 clones were apparently localized on chromosome 1 (D_5_01) of *G. raimondii* as well as on chromosomes D_h_01 and A_h_01of *G. hirsutum*. And also, the relationship of the 2 BAC clones positions in different chromosomes has been analyzed subsequently.

## Results

### Construction of cytogenetic map of *G. herbaceum* chromosome 1(A_1_01)

**Figure 1:**
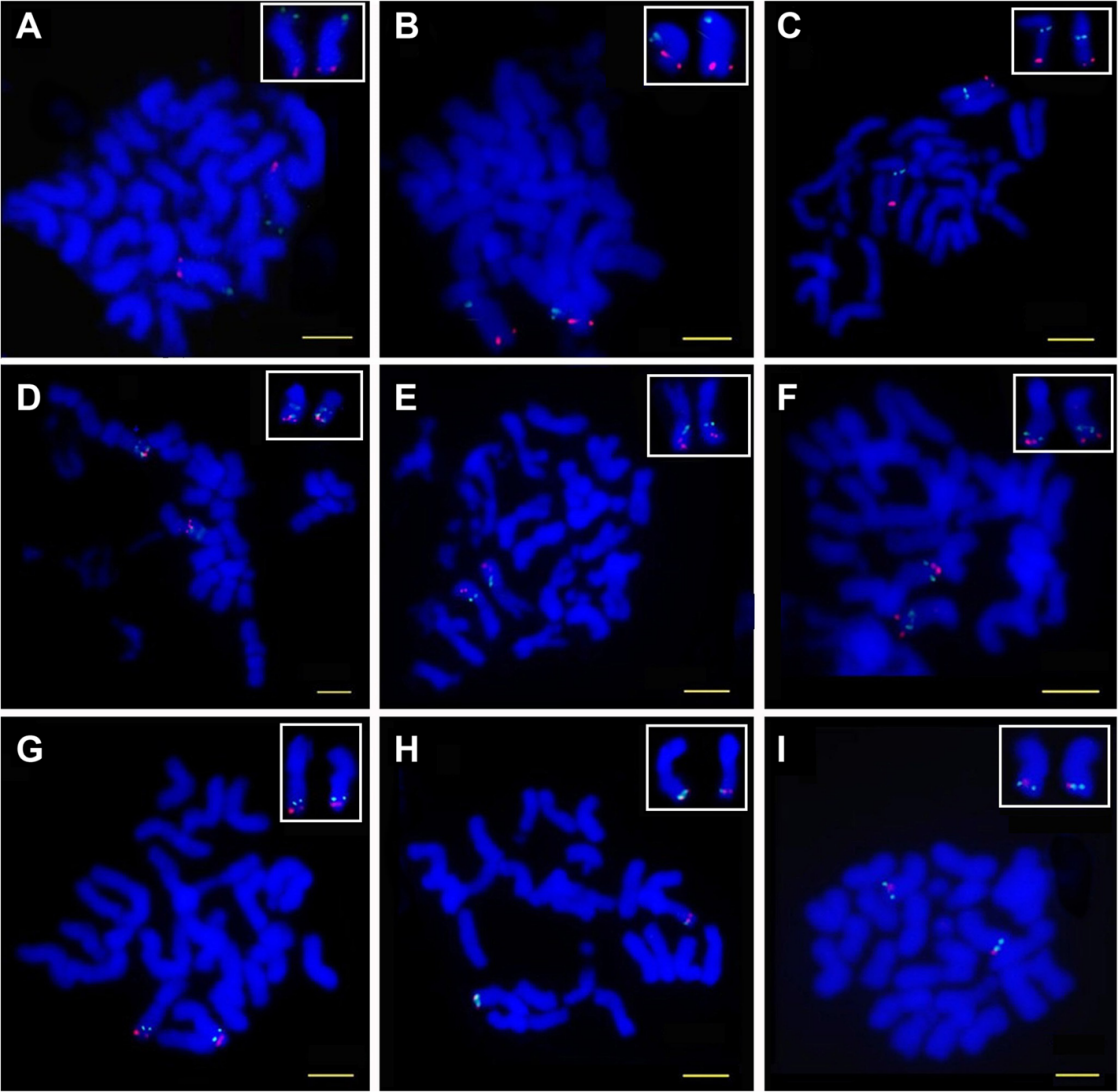
**Dual-FISH of** ***G. herbaceum*** **chromosomes with positive BAC clones (green) and chromosome 1 (A**_**1**_ **01) specific BAC clone 52D06 (BNL3580, red) as probe while counterstained with DAPI.** Bar = 5 μm. **A**: SSR marker NAU2015 (BAC clone 305A19), **B**: SSR marker NAU2474 (BAC clone144E4). **C**: SSR marker BNL2921 (BAC clone 260J3). **D**: SSR marker NAU2285 (BAC clone 263K18). **E**: SSR marker HAU076 (BAC clone 378J7). **F**: SSR marker TMB0062 (BAC clone 423C18). **G**: SSR marker NAU4891 (BAC clone 216B15). **H**: SSR marker NAU4044 (BAC clone 400L15). **I**: SSR marker NAU3135 (BAC clone 85P13).

**Figure 2:**
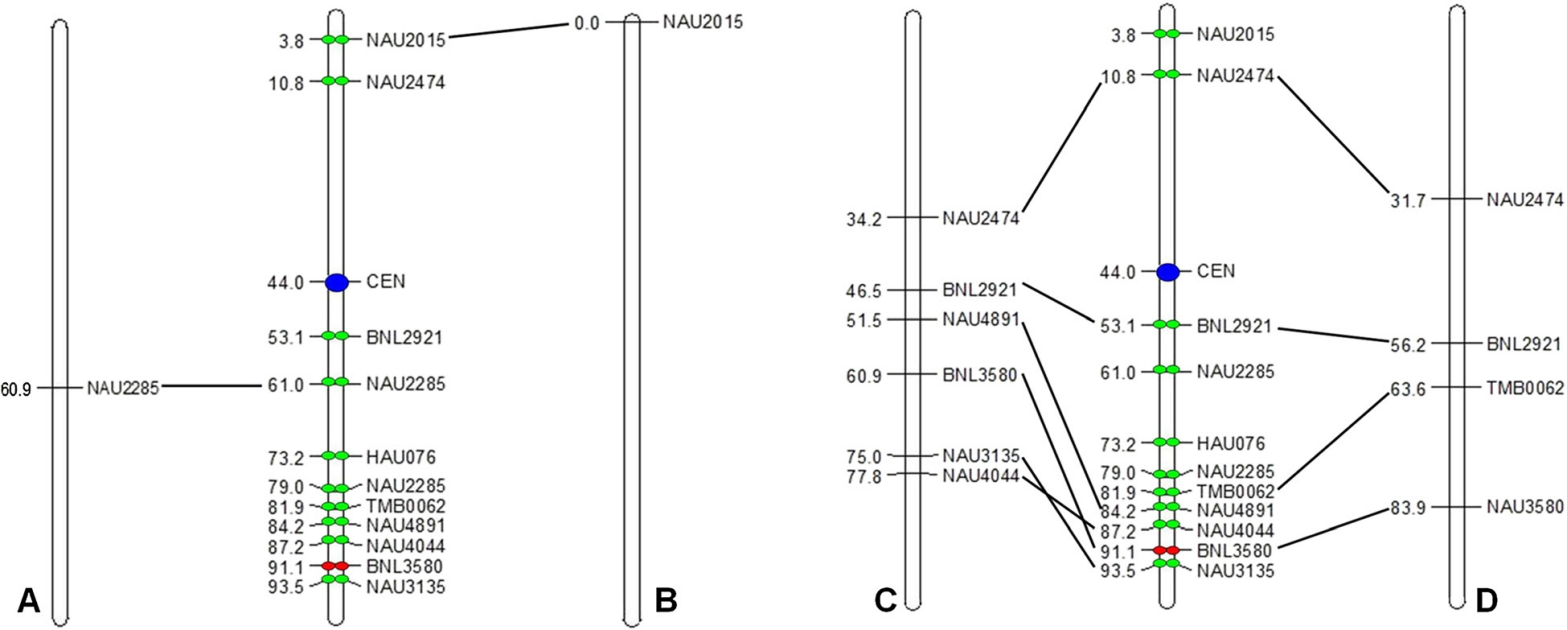
**Integration of genetic and cytogenetic maps of chromosome 1.** Ideograms of cytogenetic map showed cytogenetic locations of BAC clones and the centromere. Numbers in each map indicated the RMPs of FISH-mapped loci. **A**, **B**, **C**, and **D** are four different genetic maps. **A**: Han et al. [43] Theor Appl Genet BMC Gnomics. **B**: Ma et al. [44] G3 (Bethesda) J Integr Plant Biol. **C**: Zhao et al. [45] BMC Gnomics. **D**: Yu et al. [46] G3 (Bethesda).

To construct a cytogenetic map of *G. herbaceum* chromosome 1(A_1_01), Pima 90–53 BAC library was screened using sixteen SSR markers. The SSR markers were selected from five genetic linkage maps and used to screen the BAC library. A total of 47 positive BAC clones were identified (Table 1). The chromosome-specific BAC clone 52D06 was used to identify chromosome 1(A_1_01) of *G. herbaceum* [41,42], and all positive BAC clones of 16 SSR markers were selected for FISH mapping. Clones of 6 SSR markers which showed repetitive FISH signals in mitotic metaphase were discarded and clones of one SSR marker which showed strong signals on other chromosomes but not on chromosome 1 were also discarded (data not shown). The remaining clones of nine SSR markers with unique clear hybridization signals (Figure 1) were used for FISH mapping. Seven of them were localized on the long arm and the other two were localized on the short arm. The FISH signals of each BAC clone from more than 10 cells with clear chromosome spreads were measured and the relative positions of FISH signals were computed. More than 10 cells with clear chromosome spreads were chosen to distinguish the position of the centromere and to compute the exact cytogenetic position of the centromere. The data was analyzed to construct the cytogenetic map of *G. herbaceum* chromosome 1(A_1_01) (Figure 2).

**Table 1:** **Screened clones of Pima 90–53 BAC library**

**SSR markers**	**Screened clones from BAC library**
NAU1215	300N10
CIR342	268E2; 268K2
NAU1023	311A4; 311A11
NAU2285	328O10; 263K18
MUSS211	184B22
NAU2015	305A19
NAU3135	85P13; 377G4; 377H5; 247P16; 247P17; 325M9; 325M10
NAU4044	400L15
NAU4891	81B19; 81E20; 171I16
NAU3022	30A18; 106P24
NAU3384	328L13
NAU5100	389I13; 389L19; 389J15; 376M12; 311M1; 311M2
NAU2474	144E4; 165B11
BNL2921	260J3; 400L3; 400P6
TMB0062	298N21; 403A13; 423C18; 423C19; 424A12
HAU076	249G3; 249G4; 249I5; 325N10; 378J7; 398J5; 398H5; 249G5

### Integration and analysis of clone positions across maps

In order to analyze the relationship between genetic linkage maps and the constructed cytogenetic map, the composite alignment was constructed to compare the FISH map directly to the genetic linkage maps of *G. hirsutum* ((AD)_1_) and *G. arboreum* (A_2_) chromosome 1 (Figure 2). The alignment provided a global view of the relationship between the genetic positions of the SSR markers and the cytogenetic positions of the BAC clones anchored by corresponding SSR markers.

The comparative analysis showed that the order of most selected marker-anchored BAC clones was the same as in the linkage map, except three BAC clones anchored by markers NAU3135, BNL3580 and NAU4044 appeared different positions. These three BAC clones were very tight on the cytogenetic map, but the positions of corresponding SSR markers of them were visibly different from those on the genetic map C. Two BAC clones 305A19 and 260J3 anchored by SSR markers NAU2015 and BNL2921 respectively had good corresponding locations between genetic maps and the cytogenetic map. But the locations of other BAC clones and their corresponding markers had obvious discrepancies, especially BAC clone 216B15 anchored by NAU4891 showed a maximum difference of 32.7 RMP units. BAC clone 85P13 anchored by NAU3135 and BAC clone 426C18 anchored by TMB0062 expressed a difference of 18.5 RMP units and 18.3 RMP units, respectively. The positions of NAU2474 on genetic map A and B had little difference about 22 RMP units when compared to the positions on the cytogenetic map. The BAC clone anchored by SSR marker NAU2285 showed two clear signals on chromosome 1(A_1_01), and the location of one signal was concordant with marker position in the corresponding genetic map. SSR marker HAU076 is in a short linkage group which only has two markers on chromosome 1(A_h_01) according to genetic map E [47], here its corresponding BAC clone was localized on chromosome 1(A_1_01) and its exact physical position was obtained afterwards.

### Two clones on chromosome A_1_01 were localized on chromosome D_5_01, A_h_01 and D_h_01

**Figure 3:**
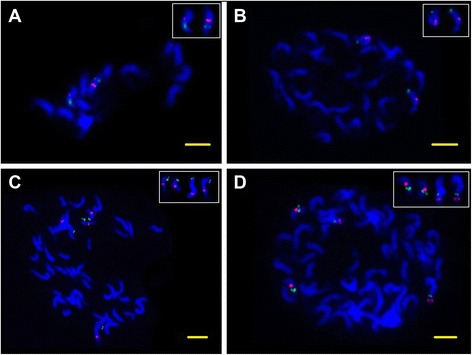
**Dual-FISH with positive BAC clones (green) and chromosome specific BAC clones (red) as probe while chromosomes were counterstained with DAPI.** Bar = 5um. **A**: BAC clone 216B15 tagged by SSR marker NAU4891 (green) and chromosome D_5_01 specific BAC clone tagged by SSR marker BNL3902 (red), *G. raimondii*. **B**: BAC clone 305A19 tagged by SSR marker NAU2015 (green) and chromosome D_5_01 specific BAC clone tagged by SSR marker BNL3902 (red), *G. raimondii.* **C**: BAC clone 305A19 tagged by SSR marker NAU2015 (green) and chromosome D_h_01, A_h_01 specific BAC clones tagged by SSR marker BNL3902, BNL3580 (red), *G. hirsutum.* **D**: BAC clone 216B15 tagged by SSR marker NAU4891 (green) and chromosome D_h_01, A_h_01 specific BAC clones tagged by SSR marker BNL3902, BNL3580 (red), *G. hirsutum.*

Chromosome-specific BAC clone 389K13 and 48F11 were used to identify chromosome 1(D_5_01) of *G. raimondii* and chromosome 15(D_h_01) of *G. hirsutum*, respectively [41,48]. Chromosome-specific BAC clone 52D06 was used to identify chromosome 1(A_h_01) of *G. hirsutum* [41]. All BAC clones of nine SSR markers distributed on chromosome 1(A_1_01) of *G. herbaceum* were selected for FISH mapping. Clones of seven SSR markers either showed repetitive FISH signals or no FISH signal in mitotic metaphase (data not shown). The remaining clones of two SSR markers showed unique clear hybridization signals on chromosome D_5_01 (Figure 3). Clone 305A19 anchored by NAU2015 was localized in the long arm and clone 216B15 anchored by NAU4891 was localized in the short arm. We measured the FISH signals of each BAC clone from more than 10 cells with clear chromosome spreads and then computed the relative position of FISH signals. The RMP unit of clone 305A19 anchored by NAU2015 is 1.8 RMP, while clone 216B15 anchored by NAU4891 is 76.5 RMP. More than 10 cells with clear chromosome spreads were chosen to distinguish the position of the centromere and the exact cytogenetic position of the centromere was computed to be 45.2 RMP. The two clones were also localized on D_h_01 and A_h_01 chromosomes of *G. hirsutum* The positions of the two clones on D_h_01 chromosome were computed to be 1.0 RMP (clone 305A19 anchored by NAU2015) and 76.0 RMP (clone 216B15 anchored by NAU4891),respectively. Whereas the positions of the two clones on A_h_01 chromosome were 1.5 RMP and 82.5 RMP,while the positions of centromere of chromosomes D_h_01 and A_h_01 were computed to be 46.5 RMP and 45.0 RMP, respectively.

### Comparison and analysis of clone positions across different chromosomes

**Figure 4:**
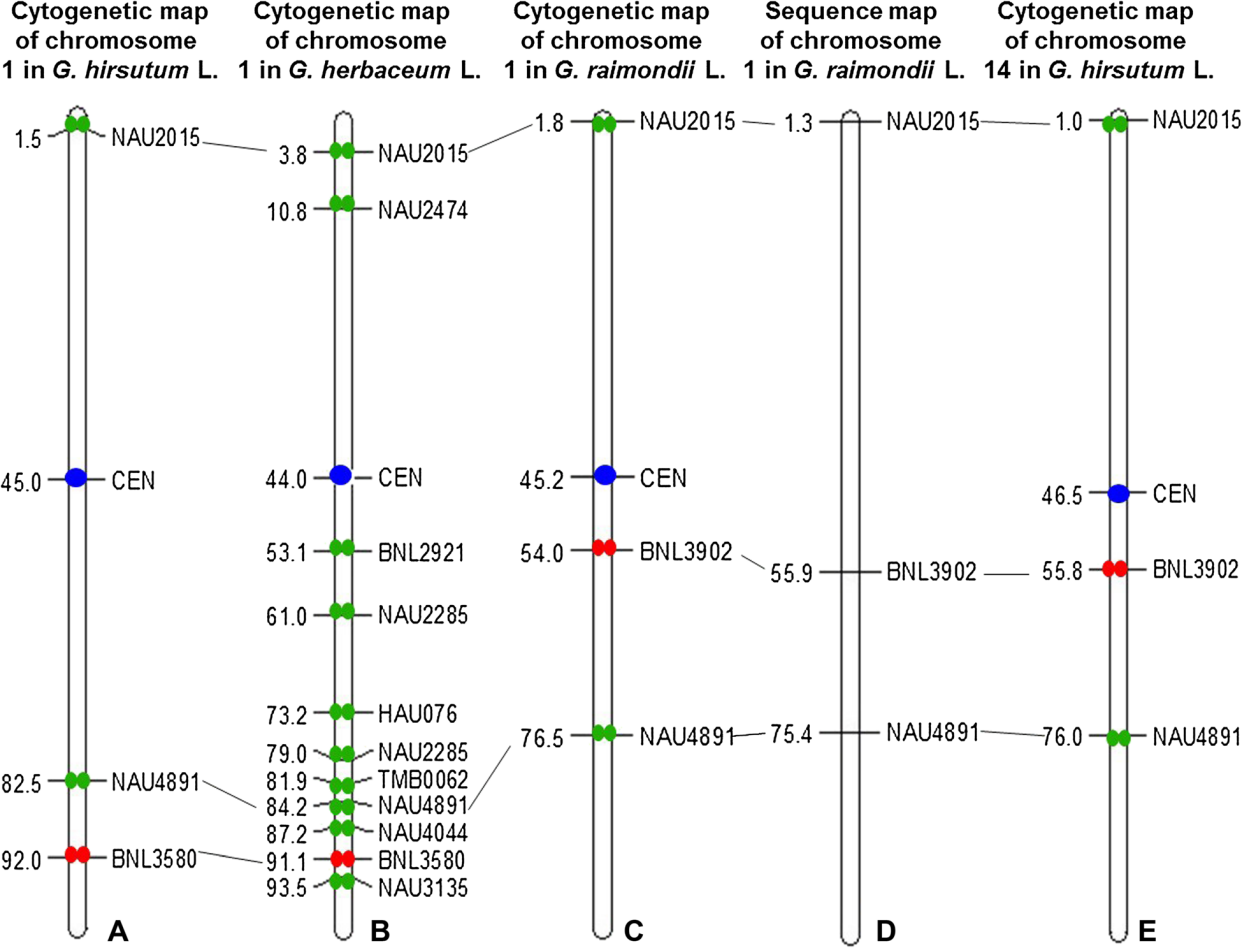
**Comparison of different maps.** Ideograms of cytogenetic maps showed cytogenetic locations of BAC clones and the centromere. Numbers in each map indicated the RMPs of FISH-mapped loci. **A**: Cytogenetic map of chromosome 1(At01) in *G. hirsutum.* **B**: Cytogenetic map of chromosome 1(A_1_01) in *G. herbaceum.* **C**: Cytogenetic map of chromosome 1(D_5_01) in *G. raimondii.* **D**: Sequence map of chromosome 1(A_5_01) in *G. herbaceum.* **E**: Cytogenetic map of chromosome 14(Dt01) in *G. hirsutum.*

By analyzing the relationship between different maps, a whole insight of chromosome structure can be displayed. And by comparing maps of homoeologous chromosomes, well conserved regions between homoeologous chromosomes can be detected. Here the positions of the two clones were compared across different chromosomes (Figure 4).

The comparative analysis showed that the order of the three marker-anchored (NAU2015, NAU4891, BNL3902) BAC clones on chromosome D_5_01 was same as in the whole-genome DNA marker map, while the distance between the markers has little discrepancies. The positions of BAC clones tagged SSR markers NAU2015 and NAU4891, respectively on D_5_01 chromosome were almost same as that on A_1_01 chromosome, except NAU7891 showed a discrepancy of 7.7 RMP. The positions of clones on chromosome A_h_01 showed a little difference from on chromosome A_1_01. And the situation was the same when comparing the positions on chromosome D_5_01 and D_h_01, respectively.

## Discussion

### Centromere positions of chromosome A_1_01, D_5_01, A_h_01 and D_h_01

The acquisition of the locations of centromeres in different chromosomes will be helpful to the study of their structure. In our research more than 10 cells were chosen with clear chromosome spreads to distinguish the positions of the centromeres of different chromosomes. And of these ten SSR markers used in this research, the centromere position was expressed as follows: *G. herbaceum* chromosome 1(A_1_01) was located between SSR marker NAU2474 and BNL2921, BNL2921 is the nearest SSR marker to the centromere. As crossovers are always low at the region near the centromere, the near markers between the two markers in genetic maps may be considered physically far apart.

### Construction of cytogenetic map for *G. herbaceum* chromosome 1 (A_1_01)

Cytogenetic map can provide information on the structure and evolution of genomes [49]. It can compensate for the disadvantages of genetic map which based on recombination frequencies that vary widely in relation to physical distances. However, few researches on cytogenetic mapping of cotton have been reported. To our knowledge, the cytogenetic map of *G. herbaceum* chromosome 1(A_1_01) reported here is the first case. The cytogenetic map including the ten BAC clones are all anchored by SSR markers. Among the ten BAC clones, eight of them were localized on the long arm, and two of them were localized on the short arm. As reported in the documentation, using conserved genes or clones in related species have proved a successful strategy in examining genome structures and relationships and predicting genes locations and DNA markers [21]. So the ten BAC clones in this article also can be used in the related species, *Theobroma cacao* even from which *Gossypium* was diverged 18–58 million years ago [50], in order to help study the relationship between these species. As the resolution of metaphase FISH is limited and highly repetitive regions which may not be represented in the BAC library exist, the coverage of the BAC map is still thought uncompleted. However, this will not influence on the cytogenetic map be used to provide a solid theoretical foundation and as an useful method to analyze the structure of *G. herbaceum* chromosome 1(A_1_01). At the same time, a reliable backbone was built up to guide in sequencing the *G. herbaceum* chromosome 1(A_1_01).

### Integration of genetic linkage maps and cytogenetic map

Genetic linkage map can provide some valuable information of the genome, but they can only provide little information about the exact cytogenetic locations of markers and the distance between them. In the present work, a cytogenetic map of *G. herbaceum* chromosome 1(A_1_01) was constructed and was shown integrated with three genetic maps of *G. hirsutum* and one genetic map of *G. arboretum,* using a standardized map unit-relative map position (RMP). It is thought exciting that this integration of maps can provide new insights into structure and organization of *G. herbaceum*.

The comparative map alignments showed that the distance of marker NAU2474 and BNL2921 on genetic map C was 12.3 RMP (16.3 cM) but on the cytogenetic map was 42.3 RMP. This phenomenon also happened between other markers (BNL2921 and TMB0062, BNL2921 and NAU4891). The discrepancies between genetic and cytogenetic distances may be because of the suppression of genetic recombination. The same observations were reported in many other plant including maize [51], wheat [52], barley [53], and sorghum [54,55]. In particular chromosomes, the distance between two markers which is more than 50% in genetic map can be very near in cytogenetic map because of the suppression of genetic recombination [53-55]. The order of positions of Marker NAU3135 BNL3580 and NAU4044 in the cytogenetic FISH map are different from the order in the genetic map C. Marker NAU3135 and NAU4044 on genetic map C were very tightly linked. The discrepancies in markers order might indicate small chromosome rearrangements, but it may also be due to the insufficient resolution of the FISH cytogenetic map in specific regions of the chromosome. The BAC clone tagged SSR marker NAU2285 showed 2 clear signals on chromosome 1(A_1_01), the location of one of the signals was well corresponding with its location on genetic maps. This may indicate that the conversed region has a translocation within chromosome 1(A_1_01). Based on the research work of Zhang [47], HAU076 is in a short linkage group that has only two markers on chromosome 1(A_h_01). In our study, its corresponding BAC clone 378J7 was localized cytogenetically on chromosome 1(A_1_01). The location of the SSR marker will be very useful for cotton genome organization architecture.

### Two SSR markers first localized on chromosome D_5_01 and D_h_01

Although more than 30 genetic maps of cotton had been constructed [56], most of them used different mapping populations with different population sizes. As a result, the genetic markers were often mapped at different genetic positions, even at different chromosomes, in different maps. So this makes it very difficult to study gene distribution, chromosome evolution, and map-based cloning between different populations [57]. The cytogenetic map is known inherently informative as it can represent direct positions of location chromosomes. It can help to resolve the locations of markers that couldn’t be resolved on genetic linkage maps, especially markers linked closely with very low rate of recombination chromosome sections. The location of two SSR markers which had never been included on chromosome D_5_01 or D_h_01 in any genetic map by using BAC-FISH method displayed here demonstrated to be a good example. In another word, the location of the two SSR markers can not only consummate genetic maps of D_5_01 and D_h_01, but also means the chromosome section which linked closely to the two SSR markers, may infer a very low rate of recombination. The first location of the two SSR markers on chromosome D_5_01 and D_h_01 will facilitate the whole-genome physical alignment, sequencing, and mapping of genes for cotton improvement.

### Two conserved regions detected between chromosomes D_5_01 (D_h_01) and A_1_01 (A_h_01)

It is known that the allotetraploid cotton contains two sub-genomes originated from the related ancestor species with nearly two fold sizes difference. The comparison analysis between homoeologous loci and chromosomes in tetraploid cotton showed that most duplicated genes in allopolyploid cotton evolved independently of each other [58], and some regions were highly conserved [5,59]. In the research of Wang et al. [11], ten of the eleven BAC clones which were localized on chromosome A_h_12 also showed signals on chromosome D_h_12; fourteen of the twenty BAC clones which were localized on chromosome D_h_12 also showed signals on chromosome A_h_12, suggesting that the majority of this pair of homoeologous chromosomes remains conserved and homologous after polyploidization occurrence. In this paper, nine BAC clones from cytogenetic map of *G. herbaceum* chromosome A_1_01 were used to hybrid chromosomes of *G. raimondii*, as a result two of them were localized on chromosome D_5_01. In addition, the two clones were also tested on chromosome A_h_01 and D_h_01 of *G. hirsutum*, showing the same positions as that on chromosome A_1_01 and D_5_01. But other seven BAC clones were not localized on chromosome D_5_01 or D_h_01. This may suggest that most parts of the pair of homologous chromosomes were not conserved.

## Conclusion

Cotton is an excellent system for the study of genome evolution and polyploidization. However, the cytology study on cotton is far behind other leading crops. Using BAC-FISH presented here, individual BAC clones all anchored by SSR markers were accurately localized on chromosomes. Two markers perhaps were first mapped on chromosome D_5_01 and D_h_01, and the conserved regions tagged by the two markers were detected between D_5_01 (D_h_01) and A_1_01 (A_h_01) chromosomes. Development of a clone-based cytogenetic map seen in the present work may also offer a resource to accelerate discovery of polymorphisms within and between breeds. Integration of genetic and cytogenetic maps not only verifies the quality of the four genetic maps but also provides important information for cotton breeding and evolution.

## Methods

### Plant materials and BAC library

The plant materials (*G. herbaceum*, accession name is Zhongcao-1; *G. raimondii*, accession name is D5-2; and *G. hirsutum*, accession name is CCRI-12) were obtained from National Wild Cotton Nursery in Hainan Island, China, sponsored and owned by the Institute of Cotton Research of Chinese Academy of Chinese Academy of Agricultural Sciences (CRI-CAAS). They are also conserved in the greenhouse at CRI-CAAS’ headquarter in Anyang City, Henan Province, China.

Pima 90–53 BAC library which was screened in this paper was kindly provided by Prof. Zhiying Ma (Hebei Agricultural University, China).

Sixteen SSR markers which were well-distributed on chromosome 1 of *G. hirsutum* (AD)_1_ and *G. arboreum* (A_2_) were selected from five genetic maps (Table 2, named as A, B, C, D, E for convenience) [43-47]. Chromosome-specific BAC clones [41,42] to identify the individual chromosomes were kindly provided by Prof. Tianzhen Zhang (Nanjing Agricultural University, China). D_5_01 Chromosome-specific BAC clone was screened by Qin [48].

**Table 2:** **SSR markers and their genetic maps**

**SSR marker**	**Genetic map of cotton**
NAU1215	Genetic map A (*Gossypium hirsutum*): Han et al. [43] Theor Appl Genet
CIR342	Genetic map A (*Gossypium hirsutum*): Han et al. [43] Theor Appl Genet
NAU1023	Genetic map A (*Gossypium hirsutum*): Han et al. [43] Theor Appl Genet
NAU2285	Genetic map A (*Gossypium hirsutum*): Han et al. [43] Theor Appl Genet
MUSS211	Genetic map B (*Gossypium arboreum*): Ma et al. [44] J Integr Plant Biol
NAU2015	Genetic map B (*Gossypium arboreum*): Ma et al. [44] J Integr Plant Biol
NAU3135	Genetic map C (*Gossypium hirsutum*): Zhao et al. [45] BMC Gnomics
NAU4044	Genetic map C (*Gossypium hirsutum*): Zhao et al. [45] BMC Gnomics
NAU4891	Genetic map C (*Gossypium hirsutum*): Zhao et al. [45] BMC Gnomics
NAU3022	Genetic map C (*Gossypium hirsutum*): Zhao et al. [45] BMC Gnomics
NAU3384	Genetic map C (*Gossypium hirsutum*): Zhao et al. [45] BMC Gnomics
NAU5100	Genetic map C (*Gossypium hirsutum*): Zhao et al. [45] BMC Gnomics
NAU2474	Genetic map D (*Gossypium hirsutum*): Yu et al. [46] G3 (Bethesda)
BNL2921	Genetic map D (*Gossypium hirsutum*): Yu et al. [46] G3 (Bethesda)
TMB0062	Genetic map D (*Gossypium hirsutum*): Yu et al. [46] G3 (Bethesda)
HAU076	Genetic map E (*Gossypium hirsutum*): Zhang et al. [47] Genome

### DNA probes preparation

The probes BAC DNA were isolated by using a standard alkaline extraction [60]. The chromosome-specific BAC clones were labeled by standard DIG-nick translation reactions, whereas the screened chromosome-specific BAC clones were labeled with Biotin-nick translation reactions, according to the instructions of the manufacturer (Roche Diagnostics, USA).

### Chromosome preparation and FISH

Mitotic chromosomes preparation and FISH procedure were conducted using a modified protocol [61]. Biotin-labeled and digoxigenin-labeled probes were detected by avidin-fluorescein (green) and anti-digoxigenin-rhodamine (red) (Roche Diagnostics, USA), respectively. Chromosomes were counterstained by 4′,6-diamidino-2-phenylindole (DAPI) in the antifade VECTASHIELD solutions (Vector Laboratories, Burlingame, CA). For the probe-cocktail mixture, gDNA was used as block DNA. The dose of block DNA was 200 times of the chromosome-specific BAC DNA. The hybridization signals were observed using a fluorescence microscope (Leica MRA2) with a charge-coupled device (CCD) camera (Zeiss Axioskop2 plus). Final image adjustments were performed by using Adobe Photoshop CS3 software.

### Comparison of maps using standardized map unit

Different kinds of maps are constructed in different method, and their units are also different. For example, the unit of genetic maps is cM (centimorgan) while the unit of cytogenetic maps is FL (FL: the percentage of the distance from the FISH site to the end of the short arm relative to the total length of the chromosome). In order to integrate different type of maps with shared markers for a comprehensive view of genome structure, the relative map position (RMP) units were used in the present study. The RMP unit of the cytogenetic map is the percentage of the distance (μm) from the FISH signal site to the end of the short arm showed relative to the total length of the chromosome (μm) and the RMP value of the genetic map is the percentage from the genetic location (cM) of each locus along the total length (cM) of the corresponding linkage group [12]. In order to establish the exact position of each clone, hybridization signal of each BAC clone was measured in more than 10 cells and the average position was computed.

## References

[1] Jiang CX, Wright RJ, El-Zik KM, Paterson AH (1998). Polyploid formation created unique avenues for response to selection in *Gossypium* (cotton). Proc Natl Acad Sci U S A.

[2] Masterson J (1994). Stomatal size in fossil plants: evidence for polyploidy in majority of angiosperms. Science.

[3] Percival AE, Wendel JF, Stewart JM, Smith CW, Cothren JT (1999). Taxonomy and germplasm resources. Cotton: origin, history, technology, and production.

[4] Wendel JF, Brubaker CL, Seelanan T, Stewart J MD, Oosterhuis D, Heitholt JJ, Mauney JR (2010). The origin and evolution of *Gossypium*. Physiology of cotton.

[5] Grover CE, Kim HR, Wing RA, Paterson AH, Wendel JF (2004). Incongruent patterns of local and global genome size evolution in cotton. Genome Res.

[6] Grover CE, Yu Y, Wing RA, Paterson AH, Wendel JF (2008). A Phylogenetic analysis of indel dynamics in the cotton genus. Mol Biol Evol.

[7] Hawkins JS, Kim H, Nason JD, Wing RA, Wendel JF (2006). Differential lineage-specific amplification of transposable elements is responsible for genome size variation in *Gossypium*. Genome Res.

[8] Cronn RC, Small RL, Haselkorn T, Wendel JF (2002). Rapid diversification of the cotton genus (*Gossypium: Malvaceae*) revealed by analysis of sixteen nuclear and chloroplast genes. Am J Bot.

[9] Wendel JF (1989). New World tetraploid cottons contain Old World cytoplasm. Proc Natl Acad Sci U S A.

[10] Wendel JF, Schnabel A, Seelanan T (1995). An unusual ribosomal DNA sequence from *Gossypium* gossypioides reveals ancient, cryptic, intergenomic introgression. Mol Phylogenet Evol.

[11] Wang K, Guo WZ, Yang ZJ, Hu Y, Zhang WP, Zhou BL, Stelly DM, Chen ZJ, Zhang TZ (2010). Structure and size variations between 12A and 12D homoeologous chromosomes based on high-resolution cytogenetic map in allotetraploid cotton. Chromosoma.

[12] Sun JY, Zhang ZH, Zong X, Huang SW, LI ZY, Han YH (2013). A high-resolution cucumber cytogenetic map integrated with the genome assembly. BMC Genomics.

[13] Kao FI, Cheng YY, Chow TY, Chen HH, Liu SM, Cheng CH, Chung MC (2006). An integrated map of *Oryza sativa* L. chromosome 5. Theor Appl Genet.

[14] Cheng ZK, Presting GG, Buell CR, Wing RA, Jiang J (2001). High-resolution pachytene chromosome mapping of bacterial artificial chromosomes anchored by genetic markers reveals the centromere location and the distribution of genetic recombination along chromosome 10 of rice. Genetics.

[15] Desel C, Jung C, Cai DG, Kleine M, Schmidt T (2001). High-resolution mapping of YACs and the single-copy gene Hs1pro-1 on *Beta vulgaris* chromosome by multi-colour fluorescence *in situ* hybridization. Plant Mol Biol.

[16] Zhu L, Smith S, de Lange T, Seldin MF (1999). Chromosomal mapping of the tankyrase gene in human and mouse. Genomics.

[17] Tang X, De Boer JM, Van Eck HJ, Bachem C, Visser RG, De Jong H (2009). Assignment of genetic linkage maps to diploid *Solanum tuberosum* pachytene chromosomes by BAC-FISH technology. Chromosome Res.

[18] Fransz PF, Alonso-Blanco C, Liharska TB, Peeters Anton JM, Zabel P, De Jong JH (1996). High resolution physical mapping in *Arabidopsis thaliana* and tomato by fluorescence *in situ* hybridization to extended DNA fibres. Plant J.

[19] De Jong JH, Fransz PF, Zabel P (1999). High resolution FISH in plants-techniques and applications. Trends Plant Sci.

[20] Jackson SA, Cheng Z, Wang ML, Goodman HM, Jiang J (2000). Comparative fluorescence *in situ* hybridization mapping of a 431-kb *Arabidopsis thaliana* bacterial artificial chromosome contig reveals the role of chromosomal duplications in the expansion of the *Brassica rapa* genome. Genetics.

[21] Amarillo FI, Bass HW (2007). A transgenomic cytogenetic sorghum (Sorghum propinquum) bacterial artificial chromosome fluorescence in situ hybridization map of maize (Zea mays L.) pachytene chromosome 9, evidence for regions of genome hyperexpansion. Genetics.

[22] Figueroa DM, Bass HW (2012). Development of pachytene FISH maps for six maize chromosomes and their integration with other maize maps for insights into genome structure variation. Chromosome Res.

[23] Howell EC, Armstrong SJ, Barker GC, Jones GH, King GJ, Ryder CD, Kearsey MJ (2005). Cytogenetic organization of the major duplication on *Brassica oleracea* chromosome O6 revealed through fluorescence *in situ* hybridization with *Arabidopsis* and *Brassica* BAC probes. Genome.

[24] Xiong Z, Kim JS, Pires JC (2010). Integration of genetic, cytogenetic, and cytogenetic maps for *Brassica rapa* chromosome A7. Cytogenet Genome Res.

[25] Chang SB, Anderson LK, Sherman JD, Royer SM, Stack SM (2007). Predicting and testing cytogenetic locations of genetically mapped loci on tomato pachytene chromosome 1. Genetics.

[26] Koo DH, Jo SH, Bang JW, Park HM, Lee S, Choi D (2008). Integration of cytogenetic and genetic linkage maps unveils the cytogenetic architecture of tomato chromosome 2. Genetics.

[27] Szinay D, Chang SB, Khrustaleva L, Peters S, Schijlen E, Bai Y, Stiekema WJ, Van Ham RC, De Jong H, Klein Lankhorst RM (2008). High-resolution chromosome mapping of BACs using multi-colour FISH and pooled-BAC FISH as a backbone for sequencing tomato chromosome 6. Plant J.

[28] Han YH, Zhang ZH, Huang SW, Jin WW (2011). An integrated molecular cytogenetic map of *Cucumis sativus* L. chromosome 2. BMC Genet.

[29] Walling JG, Shoemaker R, Young N, Mudge J, Jackson S (2006). Chromosome level homeology in paleopolyploid soybean (*Glycine max*) revealed through integration of genetic and chromosome maps. Genetics.

[30] Wai CM, Moore PH, Paull RE, Ming R, Yu Q (2012). An integrated cytogenetic and physical map reveals unevenly distributed recombination spots along the papaya sex chromosomes. Chromosome Res.

[31] Iovene M, Wielgus SM, Simon PW, Buell CR, Jiang JM (2008). Chromatin structure and cytogenetic mapping of chromosome 6 of potato and comparative analyses with tomato. Genetics.

[32] Tang X, Szinay D, Lang C, Ramanna MS, van der Vossen EA, Datema E, Lankhorst RK, De Boer J, Peters SA, Bachem C, Stiekema W, Visser RG, De Jong H, Bai Y (2008). Crossspecies BAC-FISH painting of the tomato and potato chromosome 6 reveals undescribed chromosomal rearrangements. Genetics.

[33] Pedrosa-Harand A, Kami J, Gepts P, Geffroy V, Schweizer D (2009). Cytogenetic mapping of common bean chromosomes reveals a less compartmentalized small-genome plant species. Chromosome Res.

[34] Fonsêca A, Ferreira J, Dos Santos TR, Mosiolek M, Bellucci E, Kami J, Gepts P, Geffroy V, Schweizer D, Dos Santos KG, Pedrosa-Harand A (2010). Cytogenetic map of common bean (*Phaseolus vulgaris* L.). Chromosome Res.

[35] Kim JS, Islam-Faridi MN, Klein PE, Stelly DM, Price HJ, Klein RR, Mullet JE (2005). Comprehensive molecular cytogenetic analysis of sorghum genome architecture: distribution of euchromatin, heterochromatin, genes and recombination in comparison to rice. Genetics.

[36] Wang K, Yang ZJ, Shu CS, Hu J, Lin QY, Zhang WP, Guo WZ, Zhang TZ (2009). Higher axial-resolution and sensitivity pachytene fluorescence *in situ* hybridization protocol in tetroploid cotton. Chromosome Res.

[37] Peng RH, Zhang T, Liu F, Ling J, Wang CY, Li SH, Zhang XD, Wang YH, Wang KB (2012). Preparations of meiotic pachytene chromosomes and extended DNA fibers from cotton suitable for fluorescence *In Situ* hybridization. PLoS ONE.

[38] Gan YM, Chen D, Liu F, Wang CY, Li SH, Zhang XD, Wang YH, Peng RH, Wang KB (2011). Individual chromosome assignment and chromosomal collinearity in *Gossypium thurberi*, *G. trilobum* and D subgenome of *G. barbadense* revealed by BAC-FISH. Genes Genet Syst.

[39] Gan YM, Liu F, Peng RH, Wang CY, Li SH, Zhang XD, Wang YH, Wang KB (2012). Individual chromosome identification, chromosomal collinearity and genetic-physical integrated map in *Gossypium darwinii* and four D genome cotton species revealed by BAC-FISH. Genes Genet Syst.

[40] Wang K, Guo WZ, Zhang TZ (2007). Detection and mapping of homologous and homoeologous segments in homoeologous groups of allotetraploid cotton by BAC-FISH. BMC Genomics.

[41] Wang K, Guo WZ, Zhang TZ (2007). Development of one set of chromosome-specific microsatellite-containing BACs and their physical mapping in Gossypium hirsutum L. Theor Appl Genet.

[42] Cheng H, Gan YM, Liu F, Cai XY, Wang CY, Wang YH (2013). Individual chromosome identification in G. barbadense cv. Pima 90–53, G. herbaceum cv. Hongxing, and G. herbaceum raced africanum. Cotton Sci (In Chinese).

[43] Han ZG, Wang CB, Song XL, Guo WZ, Gou JY, Li CH (2006). Characteristics, development and mapping of Gossypium hirsutum derived EST-SSRs in allotetraploid cotton. Theor Appl Genet.

[44] Ma XX, Zhou BL, Lv YH, Guo WZ, Zhang TZ (2008). Simple sequence repeat genetic linkage maps of A-genome diploid cotton (*Gossypium arboreum*). J Integr Plant Biol.

[45] Zhao L, Lv YD, Cai CP, Tong XC, Chen XD, Zhang W (2012). Toward allotetraploid cotton genome assembly: integration of a high-density molecular genetic linkage map with DNA sequence information. BMC Genomics.

[46] Yu JZ, Kohel RJ, Fang DD, Cho J, Van Deynze A, Ulloa M (2012). A high-density simple sequence repeat and single nucleotide polymorphism genetic map of the tetraploid cotton genome. G3 (Bethesda).

[47] Zhang YX, Lin ZX, Xia QZ, Zhang MJ, Zhang XL (2008). Characteristics and analysis of simple sequence repeats in the cotton genome based on a linkage map constructed from a BC_1_ population between *Gossypium hirsutum* and *G. barbadense*. Genome.

[48] Qin Q, Liu F, Gan YM, Wang CY, Wang YH, Cai XY (2013). Screening and positioning of three chromosome-specific BAC clones in *Gossypium raimondii*. Cotton Sci (In Chinese).

[49] González J, Nefedov M, Bosdet I, Casals F, Calvete O, Delprat A (2005). A BAC-based physical map of the *Drosophila buzzatii* genome. Genome Res.

[50] Li FG, Fan GY, Wang KB, Sun FM, Yuan YL, Song GL (2014). Genome sequence of the cultivated cotton *Gossypium arboretum*. Nat Genet.

[51] Wang CJ, Harper L, Cande WZ (2006). High-resolution single-copy gene fluorescence *in situ* hybridization and its use in the construction of a cytogenetic map of maize chromosome 9. Plant Cell.

[52] Gill KS, Gill BS, Endo TR, Taylor T (1996). Identification and high density mapping of gene-rich regions in chromosome group 1 of wheat. Genetics.

[53] Kunzel G, Korzun L, Meister A (2000). Cytologically integrated cytogenetic restriction fragment length polymorphism maps for the barley genome based on translocation breakpoints. Genetics.

[54] Islam-Faridi MN, Childs KL, Klein PE, Hodnett G, Menz MA, Klein RR (2002). A molecular cytogenetic map of sorghum chromosome 1 Fluorescence *in situ* hybridization analysis with mapped bacterial artificial chromosomes. Genetics.

[55] Kim JS, Klein PE, Klein RR, Price HJ, Mullet JE, Stelly DM (2005). Molecular cytogenetic maps of Sorghum linkage groups 2 and 8. Genetics.

[56] Wang ZN, Zhang D, Wang XY, Tan X, Guo H, Paterson AH (2013). A whole-genome DNA marker map for cotton based on the D-genome sequence of *Gossypium raimondii* L. G3 (Bethesda).

[57] Xu ZY, Kohel RJ, Song GL, Cho JM, Yu J, Yu SX (2008). An integrated genetic and physical map of homoeologous chromosomes 12 and 26 in Upland cotton (*G. hirsutum* L.). BMC Genomics.

[58] Cronn RC, Small RL, Wendel JF (1999). Duplicated genes evolve independently after Polyploid formation in cotton. Proc Natl Acad Sci U S A.

[59] Grover CE, Kim HR, Wing RA, Paterson AH, Wendel JF (2007). Microcolinearity and genome evolution in the *AdhA* region of diploid and polyploid cotton (*Gossypium*). Plant J.

[60] Sambrook J, Russell DW (2002). Molecular cloning: a laboratory manual.

[61] Wang CY, Wang KB, Song GL, Li MX, Bie S, Li SH (2001). Protocol of cotton FISH of somatic chromosomes with rDNA as probes. Cotton Sci (In Chinese).

